# Topconfects: a package for confident effect sizes in differential expression analysis provides a more biologically useful ranked gene list

**DOI:** 10.1186/s13059-019-1674-7

**Published:** 2019-03-28

**Authors:** Paul F. Harrison, Andrew D. Pattison, David R. Powell, Traude H. Beilharz

**Affiliations:** 1 Monash Bioinformatics Platform, Monash University, Melbourne, Australia; 2 Stem Cell and Development Program, Monash Biomedicine Discovery Institute and Department of Biochemistry and Molecular Biology, Monash University, Melbourne, Australia

**Keywords:** RNA-Seq, Differential expression analysis, Confidence interval, False discovery rate, False coverage-statement rate, TREAT

## Abstract

Differential gene expression analysis may discover a set of genes too large to easily investigate, so a means of ranking genes by biological interest level is desired. *p* values are frequently abused for this purpose. As an alternative, we propose a method of ranking by confidence bounds on the log fold change, based on the previously developed TREAT test. These confidence bounds provide guaranteed false discovery rate and false coverage-statement rate control. When applied to a breast cancer dataset, the top-ranked genes by Topconfects emphasize markedly different biological processes compared to the top-ranked genes by *p* value.

**Electronic supplementary material:**

The online version of this article (10.1186/s13059-019-1674-7) contains supplementary material, which is available to authorized users.

## Background

Misunderstanding and abuse of *p* values has led to widespread debate and proposals for the adoption of alternatives [1–3]. One moderate proposal is to switch from the reporting of *p* values to the reporting of confidence intervals (CIs) [4]. This is a shift of emphasis from a dichotomous division between zero and non-zero effect size to estimating the effect size and placing confidence bounds on this estimate. CIs are based on the same underlying theory as *p* values, providing control of the type I error probability (the probability of the false rejection of a true hypothesis) [5]. For example, Cochrane ([6], section 12.4.1) uses CIs to judge whether an intervention has not just a non-zero effect but confidently a clinically useful effect. The widely used *Publication Manual of the American Psychological Association* ([7], section 2.07) recommends giving estimated effect sizes and strongly recommends that these be accompanied by CIs, with effect sizes to be given in the original units and possibly also in a standardized form such as Cohen’s *d*.

One area this shift has not yet occurred is in differential expression analysis of microarray and RNA-Seq data. Here, the effect size of interest is generally the log_2_ fold change (LFC) in the relative RNA abundance of each gene between two groups of biological samples. A possible reason is that due to the large number of genes tested, multiple testing correction is necessary in differential expression analysis in order to maintain a false discovery rate (FDR) [8]. The dependence of FDR control on the number of discoveries made makes it difficult to reconcile with the use of CIs. An alternative would be to control the family-wise error rate (FWER) using a Bonferroni correction, which has a straightforward corresponding Bonferroni correction for CIs. However, unless conclusions depend on every single CI being correct, controlling the FWER is unnecessarily strict. A final possibility is a procedure that declares a certain number of discoveries made based on some criterion and then reports false coverage-statement rate (FCR) corrected CIs for the selected genes [9]. This has been implemented in the context of differential gene expression [10], with the criterion being that the genes have non-zero differential expression with a given FDR. An appealing feature of this approach is that the confidence intervals of discovered genes at most touch but never pass through zero LFC.

Considering the current *p* value-based practice, depending on the nature of the experiment, quality of the data produced, and the chosen FDR, the number of significantly differentially expressed genes discovered may range from just a few to thousands. However, a researcher may only have resources to follow up a limited number of genes, so that thousands of discoveries present a problem. Moreover, many genes that pass this statistical test for significance may not be sufficiently changed to be of biological significance. It is important therefore to have a way to rank genes by interest level.

A common default presentation of differential expression analysis results is to list genes in order of an “FDR adjusted *p* value.” The researcher may choose a cutoff value for this adjusted *p* value, producing a set of genes with that FDR. This then gives the researcher a means to select as many genes as they are able to further investigate: read down the list until the desired number of genes is obtained. However, statistical significance is not the same as a biologically meaningful effect size. It may be that in order to obtain a manageable set of genes by this method, the researcher chooses a far smaller FDR than they actually require. Genes may as easily be chosen by this method for low biological and technical variation as for a large LFC.

A temptation is to select a set of genes by a reasonable FDR threshold and then apply a further ad hoc filtering step to obtain a reasonably sized subset. It is a common practice to perform such filtering on the basis of estimated LFC, but better might be to use the inner end of a confidence interval. However this is done, no statistical guarantee about the LFCs is given beyond that that they are non-zero. If FCR-corrected confidence intervals were used to select the subset, these would also no longer be valid for that subset.

McCarthy and Smyth [11] propose a principled solution to the problem of too many discoveries with their TREAT method. The researcher nominates a minimum LFC effect size of interest. The TREAT method finds genes with a magnitude of effect size larger than this. Again, the researcher is presented with a list in order of adjusted *p* value and may make the final choice of FDR. However, how to choose the minimum effect size with TREAT is not necessarily obvious. On the other hand, the researcher may well be able to nominate an acceptable FDR (5% is a common choice).

Therefore, we describe here a new approach to the presentation of TREAT results in which the FDR is fixed, and genes are presented in order of a quantity we call the “confident effect size” or “confect.” If a set of genes is chosen having a magnitude of confect greater than or equal to some amount, we guarantee with the given FDR that those genes will have a true LFC magnitude greater than that chosen amount. The researcher is then easily able to choose a desired effect size of interest to follow up and is never presented with unreasonably small adjusted *p* values. Furthermore, once a set of genes is chosen, the confect values provide confidence bounds with a controlled FCR. This form of presentation is “test inversion,” converting hypothesis testing into a confidence bound calculation, however with a novel feature being the incorporation of FDR control. The confect ranking solves two problems at once, giving confidence bounds with an appropriate level of multiple testing correction and simultaneously providing a ranking of genes by confident effect size.

We show using synthetic data that the confect ranking method scales across experiment sizes. The method is then applied to a cancer dataset, which has a high degree of heteroscedasticity between genes. The confect ranking method, as compared to the *p* value ranking method, leads to a markedly different emphasis on affected biological processes.

## Results

### Confect ranking matches or outperforms alternative ranking methods in simulated data

**Fig. 1:**
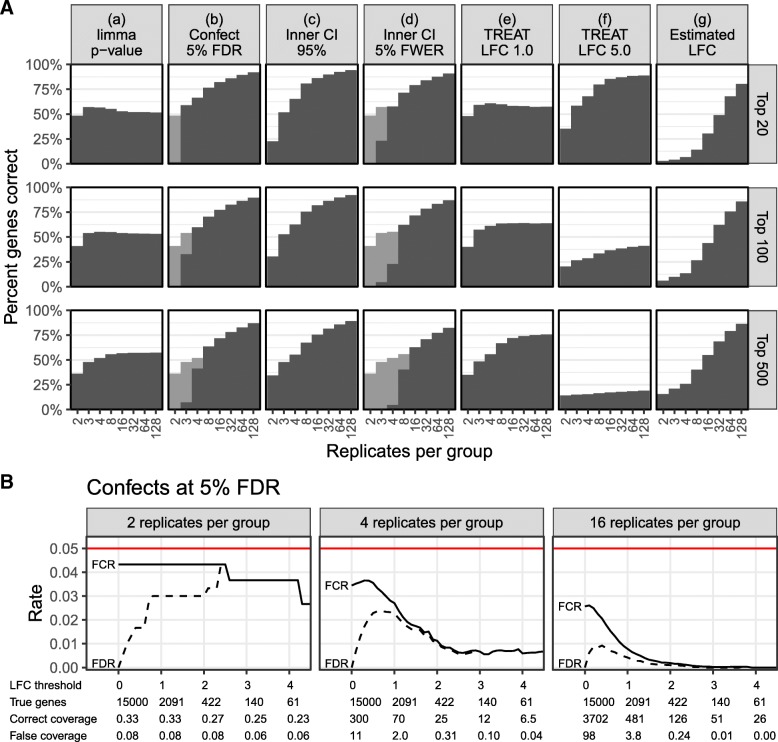
Results of simulation 1. **a** Proportion of top genes correct by various ranking methods in the top 20, 100, and 500 genes. Where genes were correct only because the ranking method fell back to ranking by limma *p* value, this is shown in gray. **b** Achieved FCR and FDR for different LFC thresholds by the confect method for a target FDR or 5%. Below the graphs, “LFC threshold” is the threshold used to select a set of genes; “True genes” is the number of genes with LFC truly exceeding this threshold. “Correct coverage” is the number of genes discovered and with true LFC correctly covered by the confidence bound. “False coverage” is the number of genes discovered but with true LFC outside the confidence bound. The sum of these last two numbers is the total number of discoveries at that threshold. All results are averaged over 100 simulations

**Fig. 2:**
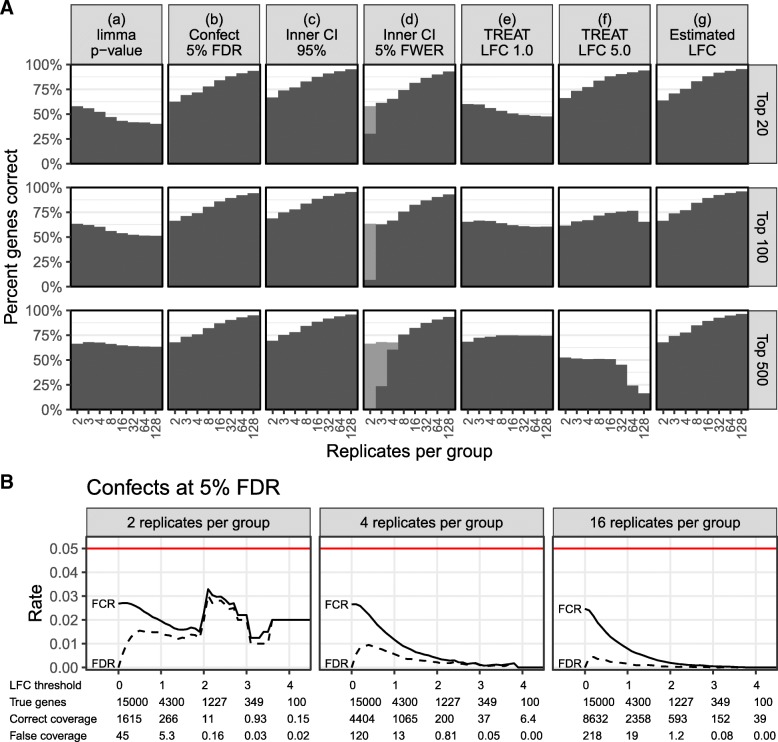
**a**, **b** Results of simulation 2. The layout is the same as in Fig. 1

To test the performance of the confect ranking method against alternative ranking methods, we generated simulated datasets with between 2 and 128 replicates per group, with parameters as described in the “Methods” section. Two simulations were performed. In simulation 1, the parameters were chosen to emphasize differences between ranking methods, and in particular, the within-group variance has been made to vary greatly between genes. In simulation 2, the parameters were chosen to match the cancer dataset to be examined next. As the data is simulated, the true LFC for each gene and the correct ranking of genes by the magnitude of LFC is known, and results from different ranking methods may be compared to this true ranking. The percentage of correct genes in the top 20, 100, and 500 genes were calculated. Results from simulation 1 are shown in Fig. 1a and from simulation 2 are shown in Fig. 2a. Complete simulation inputs and results are available from [12].

Although it is probably the most common approach to the analysis of differential gene expression, *p* value-based ranking (a) did not perform well in either simulation, nor should it be expected to as *p* values are not an indication of LFC effect size.

Confect ranking at 5% FDR (b) performs well. In simulation 1 for experiments with small numbers of replicates, this is due to falling back to ranking by the *p* value for the gene having non-zero LFC (shown as a gray bar in Fig. 1).

Interestingly, the naive method of ranking based on the inner end of a CI (c) also performed well for the purpose of ranking genes. The only place it performed worse than confect ranking was in simulation 1 for small numbers of replicates. This does not however provide control over false discoveries. The inner end of a FWER-corrected CI (d) performed less well than either the confects methods (b) or the unadjusted intervals (c).

TREAT *p* value-based ranking (e, f) may be tuned to perform well when finding a certain number of top genes, but is not a good general ranking scheme. This is as expected. The point of the confect value calculation is to modify the presentation of TREAT results to correct this shortcoming.

The estimated LFC (g) performed worst in simulation 1, but performed well in simulation 2. This is because in simulation 1 some genes with very high within-group variability will have randomly had a large estimated LFC, displacing the genes with truly large LFC. In simulation 2, the differences in variability between genes are much less pronounced.

### Confect confidence bounds provide FDR and FCR control in simulated data

Selecting a set of genes with absolute confect value exceeding some threshold *e*, the resulting genes should truly exceed this absolute LFC, with controlled false discovery rate. Furthermore, the confect values when considered as confidence bounds for the selected genes should achieve a controlled false coverage-statement rate. A false coverage-statement has been made if the sign is incorrect or the magnitude of the confect is larger than the true LFC. Achieved FDR and FCR are assessed at different thresholds for the two simulations in Figs. 1b and 2b. Ideally, achieved FDR and FCR would match the nominal level of 5%, but it can be seen that they fall below this. Fewer false discoveries and coverage statements are made than is allowed, and some potential further true discoveries may have been missed. There are two major reasons for this. Firstly, the TREAT method is conservative. It must produce *p* values for the worst case of a true LFC at the edge of the null hypothesis region, but in these simulations, the actual LFCs are clustered near zero, far from the edge of the null hypothesis region. Secondly, the confidence bounds provided by confects must work with all possible thresholds, and so have a larger adjustment than is required by Benjamini and Yekutieli’s rule [9] after a specific threshold is chosen.

The performance of the confect method is compared to inner bounds from FWER-adjusted CIs and unadjusted CIs in Additional file 1: Figures S1 and S2. FWER adjustment also provides FDR and FCR control but, as can be seen, it falls even further below the 5% level. Bounds from unadjusted CIs do not provide FDR or FCR control.

### In a cancer dataset, sorting by confident effect size rather than *p* value highlights different biological pathways

To understand how ranking by confect rather than *p* value impacts the interpretation of real experimental data, we turned to tumor-normal comparisons of breast cancer patients (BRCA) within the The Cancer Genome Atlas (TCGA). With this breast cancer dataset, limma assigns low prior degrees of freedom of 3.6, indicating a high degree of heteroscedasticity: different genes have very different levels of variability. The variance moderation applied here by limma is minor in relation to the 96 residual degrees of freedom.

**Fig. 3:**
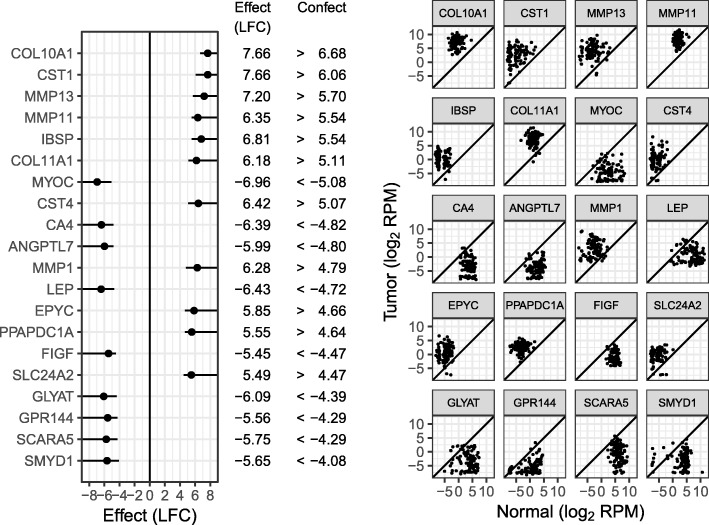
Top 20 genes by confect ranking of the breast cancer dataset at 5% FDR. For each gene, the dot shows the estimated LFC and the line shows the “confect” confidence bound. To the right, normal and tumor expression levels for all patients are shown for each listed gene

**Fig. 4:**
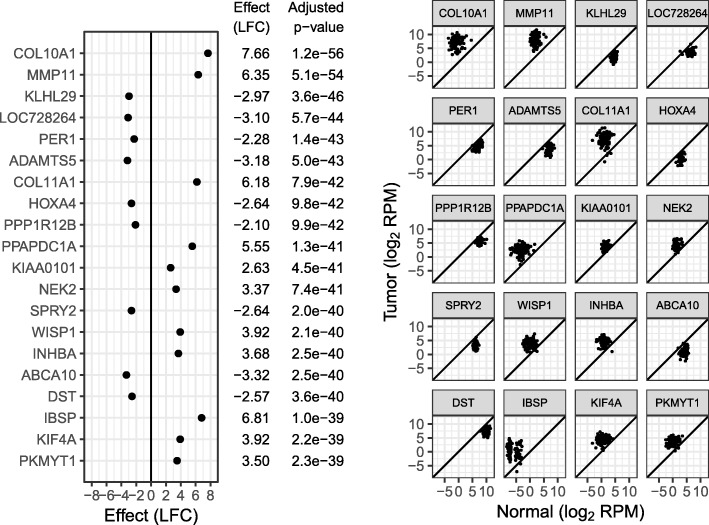
Top 20 genes by limma *p* value-based ranking of the breast cancer dataset. *p* values shown are FDR adjusted

Of the 17,932 genes tested, 13,784 are found to be differentially expressed at 5% FDR (this also means 13,784 genes are given a confect value at 5% FDR). Such a large list is of little use to a biologist prioritizing genes for further investigation. Therefore, we compared the top 20 genes ranked by confect at 5% FDR (Fig. 3) and the top 20 genes ranked by limma *p* value (Fig. 4). The full rankings are included in Additional files 2 and 3, respectively. The facetted plots to the right of the main listing in these figures show the raw data for each gene. The two methods of ranking have highlighted very different patterns of gene expression. Ranking by confect, the top genes have large LFC. The variability in LFC between patients is high in these genes; however, the confect values are also large, giving confidence that the population average LFC is truly large. Note that sets of genes at the top of the confect ranking can be obtained using the TREAT method directly. For example, the top 10 genes would be obtained using TREAT with an LFC threshold of 4.8 (the absolute confect value for the 10th gene in the ranking). However, arriving at this threshold without using confect values would require trial and error.

Examining the ranking by *p* value, the top genes may have smaller average LFCs if the LFC also has smaller variability between patients. Examples of such genes are NEK2 and KIF4A, both involved in chromosome segregation for cell division.

Gene set enrichment was searched for using the R package fgsea. There were 5182 GO Biological Process gene sets available with between 15 and 2000 genes. At 5% FDR, 453 of these gene sets are significantly enriched when ranking genes by *p* value, and 1294 are significantly enriched when ranking by confect. This is too many gene sets to reasonably examine, so the Normalized Enrichment Score effect size was used to find the top enriched gene sets. Table 1 shows the top 10 enriched gene sets for both ranking methods. For the *p* value ranking, the emphasis is on processes associated with cell division as can be expected for oncological cell transformation. For the confect ranking however, a variety of biological processes are found at the top of the list, including cell-cell signaling and blood vessel development suggestive of the tumor micro-environment. Also notable is the presence of genes involved in the extra-cellular matrix in the top 20 genes, including two collagen (COL10A1, COL11A1) and three matrix metalloproteinase genes (MMP13, MMP11, MMP1). Only three of these are seen in the top 20 genes by *p* value.

**Table 1:** Top-enriched GO biological process gene sets by NES, based on *p* value and confect rankings. Columns “Up” and “Down” are the percent significantly upregulated and downregulated genes in the cancer samples at 5% FDR

Ranking method	GO term	Description	NES	Genes	Up (%)	Down (%)
*p* value	GO:0051301	Cell division	4.81	582	54	30
	GO:0000819	Sister chromatid segregation	4.42	232	65	20
	GO:0007049	Cell cycle	4.41	1778	48	34
	GO:0022402	Cell cycle process	4.35	1309	50	32
	GO:0007059	Chromosome segregation	4.34	344	60	23
	GO:0098813	Nuclear chromosome segregation	4.18	295	60	23
	GO:0000070	Mitotic sister chromatid segregation	4.12	149	62	21
	GO:1903047	Mitotic cell cycle process	4.08	826	54	28
	GO:0000278	Mitotic cell cycle	3.99	1003	52	30
	GO:0006334	Nucleosome assembly	3.86	106	74	18
Confect	GO:0003008	System process	6.32	1472	26	52
	GO:0007186	G protein-coupled receptor signaling pathway	5.35	771	29	51
	GO:0009888	Tissue development	5.23	1686	36	43
	GO:0035295	Tube development	5.16	918	31	47
	GO:0007267	Cell-cell signaling	5.11	1481	35	45
	GO:0048646	Anatomical structure formation involved in morphogenesis	4.92	971	32	48
	GO:0001568	Blood vessel development	4.87	608	29	52
	GO:0006928	Movement of cell or subcellular component	4.85	1815	35	44
	GO:0040011	Locomotion	4.85	1578	35	45
	GO:0010469	Regulation of signaling receptor activity	4.84	413	34	47

**Fig. 5:**
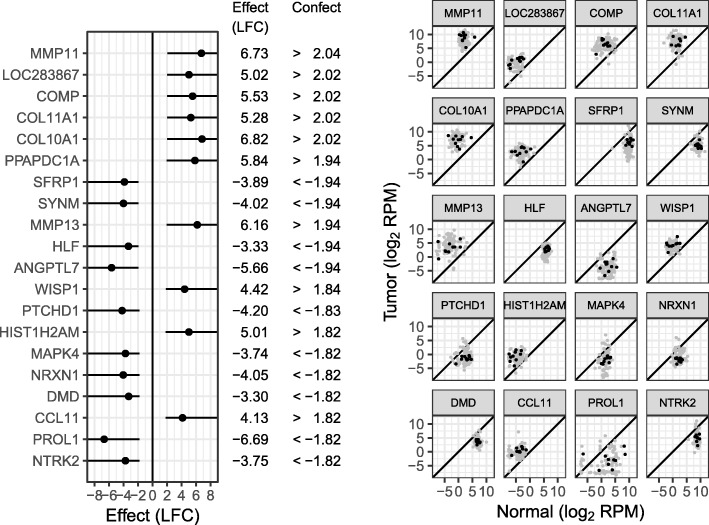
Top 20 genes by confect ranking of the breast cancer dataset at 5% FDR, using only 10 patients’ data. In the individual gene expression plots to the right, selected patients are shown as black dots and the remaining patients are shown as gray dots

Few biological experiments contain the very large number of samples present in consortia data such as the TCGA. A smaller dataset may be simulated by taking a random subset of patients. Results using a random subset of 10 patients are shown in Fig. 5 (see also Additional file 4). For the top-ranked genes, the confect values are a much smaller fraction of the effect sizes than with the full dataset. Not all of the genes with large effect sizes found in the full dataset are near the top of the list in this subset, and some genes with smaller effect sizes have been “lucky” and are highly ranked, such as COMP (jumping from 45th in the full dataset to 3rd in the subset). “Luck” of this kind is inevitable in a small dataset with this level of heteroscedasticity, and the small confect values warn that this is occurring. Similarly, by conventional *p* value-based differential expression analysis, genes in an underpowered experiment would need a combination of a large effect size and a certain amount of luck to be declared significantly DE. Also note that if TREAT were being used directly, the LFC threshold would need to be adjusted between the full dataset and the subset in order to obtain a set of genes of reasonable size. The LFC threshold in TREAT may be viewed as a threshold on the confidence bound and not the effect size itself and hence needs to be adjusted to suit the size of the experiment. The confect ranking method removes this need for parameter adjustment.

## Discussion

The effect size used here was the LFC, with the intention of finding changes in expression with a large biological effect. The confect ranking method identifies genes with confidently large LFC. This places all genes on the same scale, and this scale has meaningful units of log_2_ fold change.

Can a case for using *p* values as an effect size be made? What follows is an attempt. In fields such as psychology where the thing being measured may not have a scale with meaningful units or where there may be a number of different scales on which something may be measured, standardized effect sizes are used. Cohen’s *d* is one such standardized effect size. Cohen’s *d* is the ratio of an effect size to some appropriate standard deviation (several choices are possible [4, 13]). Applied to differential gene expression, a problem is that each gene has its own standard deviation and is therefore effectively placed on a different scale, but a situation where comparing Cohen’s *d* between genes might be appropriate would be to identify reliable prognostic biomarkers, where the interest is in genes for which the signal exceeds the background noise level. Leaving aside the use of variance moderation in limma, and when the standard deviation used is the residual standard deviation of the linear model used, Cohen’s *d* is proportional to the *t* statistic, and *p* is a monotonic function of ∣*t*∣, so *p* values can serve as a kind of standardized effect size, albeit one that is not comparable between experiments of different sizes. While the *p* values shown in Fig. 4 are meaninglessly small when considered as *p* values, they may have some meaning when considered in this way.

In the breast cancer dataset, the different ranking methods lead to an emphasis on different biological processes, both in the top-ranked genes and in downstream gene set enrichment analysis. The difference may be largely explained by the difference in ranking between Cohen’s *d* and LFC effect sizes. The common practice of using the *t* statistic for gene set enrichment tests is also effectively a choice to use Cohen’s *d*, as discussed above.

By concentrating on the top 20 genes in the cancer dataset, confidence bounds on gene LFCs were selectively reported. When unadjusted confidence bounds are selectively reported, they can become invalid, but the FCR control provided by the confects method ensures that the confidence bounds it provides can still be trusted when only top-ranked genes are selected.

limma’s TREAT method was used here as the basis of the confect calculation. The TREAT method has been extended to negative binomial generalized linear models (GLMs) and quasi-likelihood models in the edgeR R package’s glmTreat function, specifically the null = “worst.case” mode [14]. The DESeq2 R package [15] also provides a suitable test relative to a threshold for negative binomial GLMs using the result function in altHypothesis = “greaterAbs” mode. Confect calculation based on these methods is also supported by our topconfects R package.

The approach taken here has been frequentist, aside from the use of Empirical Bayes moderation of variance. FDR control has two potential Bayesian analogs. One is the “local fdr” [16, 17], the probability of the LFC being zero. This has the drawback of requiring dichotomous prior beliefs in which there is non-zero probability of the LFC being exactly zero. Earlier we noted that a significant rejection of the hypothesis that the LFC is zero also implies that the sign of the LFC is confidently known. This leads to a second Bayesian analog, the probability of having falsely called the sign of an LFC, which can be used to control the “Type S” error rate [18]. A Bayesian analog to confect values would be credible bounds on LFCs. As with local fdr and false sign probabilities, the key to this is a hierarchical model in which the prior distribution of LFCs is modeled accurately. It is not clear what the correct form of distribution of LFCs is, so this needs a statement of prior beliefs that accommodates many possibilities. As was seen here in the cancer data, different gene sets may have different distributions of LFCs, and a fully accurate statement of prior beliefs might also take this into account. DESeq2 has recently added Bayesian “False Sign Or Small” probabilities and shrunken LFC estimates based on modeling the distribution of LFCs using either the apeglm [19] or ashr [20] packages. The output of credible bounds would be a simple alternative presentation of the underlying posterior distributions here.

The FCR achieved by the method described here fell short of the nominal FCR (the confidence bounds were conservative), and a Bayesian approach may remedy this.

## Conclusions

The confect ranking method described here makes good use of any amount and quality of data. There is only one parameter, the desired FDR, for which a sensible default can be given. The resulting confect quantities are used in a similar way to FDR adjusted *p* values to select a set of genes of interest and have some similar properties. However, confect values are in the same units as the effect size (here LFC), making them easier to interpret. Comparing confect values to estimated LFC values provides feedback on whether or not an experiment was underpowered. The common practice of performing an ad hoc filtering step by estimated LFC is no longer necessary, and compared to TREAT, which provides a more principled method of filtering by LFC, even the need to provide a threshold is removed. Overall, this method of differential expression analysis has improved usability, with less expertise required in the choice of parameters and in interpretation.

## Methods

The confidence bound calculation requires as input a *p* value function *p*_*i*_(*e*) for each gene *i*, 1 ≤ *i* ≤ *n*_gene_, for a test of the null hypothesis that the absolute effect size is at most *e*. *p*_*i*_(*e*) will be a non-decreasing function of *e*. The TREAT method [11] provides a suitable *p* value function, with the effect size being LFC. The limma R package [21] provides an implementation of this in the treat function.

In the following section, TREAT will be described. Next, confidence bounds derived from TREAT are considered for a fixed significance level cutoff *α*. Finally, this is extended to FDR control, in which confidence bounds are found with a dynamic significance level cutoff.

### TREAT

Let *x*_*i*_ be the estimated LFC of the *i*th gene, and *s*_*i*_ the estimate’s standard error with *d* degrees of freedom. TREAT is a replacement for the *t* tests usually performed by the limma method of differential expression analysis [21] and makes the same adjustments to *s*_*i*_ and *d* to incorporate Empirical Bayes prior information. *e* is the LFC threshold being tested. Let *F* be the cumulative distribution function of the *t* distribution with *d* degrees of freedom. The TREAT *p* value, from [11], is

$$ {p}_i(e)=1-F\left(\frac{\left|{x}_i\right|-e}{s_i}\right)+1-F\left(\frac{\left|{x}_i\right|+e}{s_i}\right) $$

**Fig. 6:**
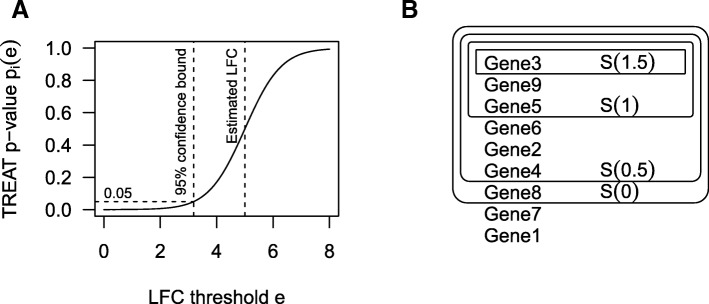
**a** TREAT *p* value (*p*_*i*_(*e*)) as a function of LFC (*e*), for *x*_*i*_ = 5,  *s*_*i*_ = 1,  *d* = 10. A 95% lower confidence bound on the effect size can be found from the *e* at which *p*_*i*_(*e*) = 0.05. **b** Illustration of ranking method. Sets *S*(*e*) are sets of genes with effect size significantly exceeding threshold *e* at some desired FDR. These sets nest, providing a ranking of genes. From this diagram, the resulting confect values would be 1.5, 1, 1, 0.5, 0.5, 0.5, and 0 and with no value given to the final two genes

An example of the shape of this function is shown in Fig. 6a. For the case of *e* = 0, the *p* value is the same as for a two-sided *t* test. We now check that this is a non-decreasing function of *e*. The derivative of *F* is the probability density function of the *t* distribution:

$$ {F}^{\prime }(t)=\frac{\Gamma \left(\frac{d+1}{2}\right)}{\sqrt{d\pi}\Gamma \left(\frac{\mathrm{d}}{2}\right)}{\left(1+\frac{t^2}{d}\right)}^{-\frac{d+1}{2}} $$

All that is needed is to observe that this is a decreasing function of *t*^2^. Now taking the derivative of *p*_*i*_(*e*) with respect to *e*,

$$ {p}_i^{\prime }(e)=\frac{1}{s_i}{F}^{\prime}\left(\frac{\left|{x}_i\right|-e}{s_i}\right)-\frac{1}{s_i}{F}^{\prime}\left(\frac{\left|{x}_i\right|+e}{s_i}\right) $$

*e* ≥ 0 and assuming *s*_*i*_ > 0, it must be that ${\left(\frac{\left|{x}_i\right|-e}{s_i}\right)}^2\le {\left(\frac{\left|{x}_i\right|+e}{s_i}\right)}^2$. Therefore, ${p}_i^{\prime }(e)\ge 0$, and therefore, *p*_*i*_(*e*) is a non-decreasing function of *e*.

### Confidence bounds from TREAT

There is a close relationship between CIs and *p* values. For example, considering the two-sided *t* test of the null hypothesis that the LFC of a gene is *e*, the null hypothesis is not rejected for values of *e* where *p*^*t* − test^(*e*) > *α*, and these values then form a 1 − *α* confidence interval. Similarly, a confidence bound can be obtained from the one-sided *t* test. This is called test inversion.

Note in particular that a significant result on a *t* test for *e* = 0 not only establishes that the LFC is non-zero, but also establishes that the sign of the LFC is known, since the corresponding confidence interval will lie either entirely above or below 0.

In the case of TREAT, the null hypothesis is that the LFC lies inside the range [−*e*, *e*]. Thus, TREAT *p* values are always larger than those from the *t* test that the LFC is 0, and a significant TREAT result determines the sign of the LFC. Taking the small liberty of considering that these two properties hold simultaneously, we view the largest *e* for which *p*_*i*_(*e*) ≤ *α* as providing a confidence bound, establishing either that the LFC is greater than *e*, or establishing that it is less than −*e*.

### Calculation of confects

Using TREAT, and making the assumption that each gene is independent of the others, a set of genes with a magnitude of effect size exceeding *e* at a given FDR *q* may be obtained using the procedure of Benjamini and Hochberg [8]. Benjamini and Hochberg’s procedure is to find the *largest* set *S*(*e*) satisfying

$$ S(e)=\left\{i:{p}_i(e)\le \frac{\mid S(e)\mid }{n_{\mathrm{gene}}}q\right\} $$

Sets for different effect sizes nest. If *e* > *e*′, then *S*(*e*) ⊆ *S*(*e*′). Genes may drop out of *S*(*e*) as *e* increases for two reasons. Firstly, *p*_*i*_(*e*) may rise above the threshold for inclusion in the set. Secondly, the threshold for inculsion in the set is a function of the size of the set ∣*S*(*e*)∣, so as the set becomes smaller, the threshold also becomes stricter. Thus, as one gene drops out several more may also need to immediately be dropped.

Let ∣*c*_*i*_∣ be the largest *e* such that *i* ∈ *S*(*e*), and let the sign of *c*_*i*_ be the actual sign of the estimated effect. We call this quantity the “confect,” for *con*fident ef*fect* size. In our implementation, when computing *c*_*i*_, we scan through a discrete set of effect sizes, by default considering *e* = 0, 0.01, 0.02, 0.03, … until *S*(*e*) is empty.

By presenting genes in order from largest to smallest ∣*c*_*i*_∣, the researcher may easily choose an effect size resulting in a set of genes *S*(*e*) of a size suitable for their purpose. It may happen that some genes have the same ∣*c*_*i*_∣, and in order to obtain a total order, we sort these by *p*_*i*_(*e*) at the first *e* for which they are not in *S*(*e*). Some genes are not a member of any set and are not given a confect. These are listed last, in order of *p*_*i*_(0) (the *p* value given by limma without using TREAT). An illustration of this method is shown in Fig. 6b.

The overall effect of this procedure is to provide a lower confidence bound on the magnitude of LFC for each gene, but with a higher level of confidence required for the larger effect sizes at the top of the list than for the smaller effect sizes lower down the list. This idea is made precise in the next section. Further, the set {*i*:| *c*_*i*_| ≥*e*} is precisely *S*(*e*) and is always at the top of the ordering.

R code implementing this procedure is provided at https://github.com/pfh/topconfects.

### Relationship to false coverage-statement rate (FCR)

If a set of genes are selected, by any selection rule, FCR controlling confidence intervals or bounds for the selected genes may be constructed using a rule described by Benjamini and Yekutieli [9]. This rule is that to ensure an FCR of *q* with *n*_selected_ genes selected, each confidence region must have coverage probability no less than $1-\frac{n_{\mathrm{selected}}}{n_{\mathrm{gene}}}q$. Based on the preceding discussion, for confect confidence bounds, this is achieved if ${p}_i\left(\left|{c}_i\right|\right)\le \frac{n_{\mathrm{selected}}}{n_{\mathrm{gene}}}q$.

Suppose a set of genes are selected based on having an absolute confect value of at least *e*. That is, they are the members of *S*(*e*) and *n*_selected_ = |*S*(*e*)|. Then, for each gene *i* in the set, we have a confect value *c*_*i*_ derived from membership in a set *S*(|*c*_*i*_|), |*c*_*i*_| ≥ *e*, and by the nesting of sets |*S*(|*c*_*i*_|)| ≤ *n*_selected_. By the definition of the members of the sets, we have

$$ {p}_i\left(\left|{c}_i\right|\right)\le \frac{\left|S\left(\left|{c}_i\right|\right)\right|}{n_{\mathrm{gene}}}q\le \frac{n_{\mathrm{selected}}}{n_{\mathrm{gene}}}q $$

Therefore, the coverage probability of each gene in the set is sufficient to control the FCR.

### Evaluation with synthetic data

Simulated data for *n*_gene_ genes is generated for two equally sized groups with *n*_rep_ samples within each group. We follow the distributional assumption of limma [21] that the gene-wise within-group variances ${\sigma}_i^2$ follow a scaled inverse chi-square distribution with degrees of freedom *d*_within_ and scale parameter *s*_within_.

$$ \frac{d_{\mathrm{within}}{s}_{\mathrm{within}}^2}{\sigma_i^2}\sim {\chi}_{d_{\mathrm{within}}}^2 $$

limma’s calculation of *p* values, both normally and with the TREAT method, do not depend on any assumption about the distribution of LFC. limma’s calculation of the posterior log-odds *B* statistic does make such assumptions, specifically that there are a set of genes that are not differentially expressed, and the ratio of LFC to *σ*_*i*_ for the differentially expressed genes follows a specific distribution. This *B* statistic is not used here. The intent of this paper is to move from the dichotomous mode of thinking associated with *p* values to the estimation mode of thinking associated with effect sizes [4], so our simulations do not assume any gene has precisely zero LFC. However, in a typical experiment, some genes are differentially expressed to a much greater extent than the majority.

Two simulations were performed, with different distributions of LFCs. “Simulation 1” investigates the scenario of large LFCs in some genes and large within-group variances in some genes, in order to best demonstrate differences between ranking methods. “Simulation 2” is a less variable simulation based on the cancer dataset described in the next section.

In simulation 1, we use a distribution of LFCs *β*_*i*_ with tails following a power law, specifically a scaled *t* distribution with *d*_between_ degrees of freedom and scaling factor *s*_between_.

$$ \frac{\beta_i}{s_{\mathrm{between}}}\sim t\left({d}_{\mathrm{between}}\right) $$

In particular, the values used for the simulation were *n*_gene_ = 15000, *n*_rep_ = 2, 3, 4, 8, 16, 32, 64 and 128, *d*_within_ = 2, *s*_within_ = 0.75, *d*_between_ = 3, and *s*_between_ = 0.5. Note in particular that *d*_within_ has been chosen to be extremely small, which will generate a highly heteroscedastic dataset, in order to emphasize differences between different ways of ranking genes. Results are averaged over 100 runs of the simulation.

In simulation 2, we approximated the distribution of LFCs and residual variances seen in the cancer dataset described in the next section. A *t* distribution was not a good fit to the LFCs in this data, and a Laplace distribution was found to be a better fit. This has exponential rather than power-law tails.

$$ {\beta}_i\sim \mathrm{Laplace}\left(0,{s}_{\mathrm{between}}\right) $$

In particular, the values used for simulation 2 were *n*_gene_ = 15000, *n*_rep_ = 2, 3, 4, 8, 16, 32, 64 and 128, *d*_within_ = 5, *s*_within_ = 0.5, and *s*_between_ = 0.8. When choosing these values, maximum likelihood estimation was tried but did not lead to a good match to the tails of the cancer dataset distributions, so these values have been manually chosen to better match the tails of these distributions (shown in Additional file 1: Figure S3). Again, *d*_within_ is an important parameter, and the higher value used here results in lower heteroscedasticity. Results are again averaged over 100 runs of the simulation.

Seven different methods of ranking genes are compared:

- Ranking by limma *p* value. Ranking by *p* value is a common default output of differential expression software.
- Confect ranking at 5% FDR. This is the proposed method, with a reasonable choice of FDR.
- Ranking by the inner end of a 95% CI. Where the CIs span zero, genes are further ranked by limma *p* value. While this does not control the FDR, its accuracy as a ranking method is of interest.
- Ranking by the inner end of a Bonferroni-corrected CI maintaining a FWER of 5%. Where the CIs span zero, genes are further ranked by limma *p* value. This is a very strict correction for multiple testing.
- Ranking by TREAT *p* value with LFC threshold 1.0. While not a general ranking by LFC effect size, this should serve to distinguish genes having LFC magnitude exceeding 1.0 from those that do not.
- Ranking by TREAT *p* value with LFC threshold 5.0.
- Ranking by the magnitude of the LFC estimated by limma. If the noise level ${\sigma}_i^2$ was uniform over all genes, this would be the ideal ranking method. The ranking methods that perform better than this one will do so based on their ability to adapt to heteroscedasticity.

### Evaluation with cancer data

RNA-Seq read counts for genes for 97 tumor-normal pairs from the TCGA BRCA breast cancer dataset were obtained from FireBrowse [22]. There was an average of 85 million reads counted per sample. The edgeR R package (version 3.22.5) was used to estimate TMM-adjusted library sizes [23]. Genes with less than an average of 0.1 reads per million (RPM) were removed from further processing. The limma function voom was then used to convert the count data to log_2_ RPM, with associated observation-level weights. The limma R package (version 3.36.5) was then used to fit linear models for each gene suitable for performing a paired-sample test for differential expression between tumor and normal samples [21], and Empirical Bayes variance moderation was applied. The method described above was then used to calculate confect values and rank genes, using a target FDR of 5%.

### Gene set enrichment

In order to better understand the biological processes emphasized by different methods of ranking genes, R package fgsea (version 1.6.0) was used to find enriched gene sets associated with Gene Ontology (GO) biological process terms [24]. fgsea implements the gene set enrichment analysis (GSEA) method, in particular the variant of the method for a pre-ranked list of genes [25]. The exponent parameter *p* is set to 0, so that the results are based purely on the ranking, and not on any associated scores. The effect size used to rank gene sets was the normalized enrichment score (NES) produced by this method. A *p* value testing whether the NES is non-zero is available from fgsea, but unfortunately no confidence interval. Gene sets containing between 15 and 2000 genes were considered. Ten thousand permutations were used when calculating *p* values.

## Additional files

Additional file 1: Additional figures. Additional figures giving more details on the simulation results. (PDF 2197 kb)

Additional file 2: Complete confect ranking of breast cancer dataset. Confect ranking of breast cancer dataset at 5% FDR. (CSV 1085 kb)

Additional file 3: Complete *p* value-based ranking of breast cancer dataset. *p* value ranking of the breast cancer dataset. (CSV 1355 kb)

Additional file 4: Confect ranking of breast cancer dataset with 10 patients. Confect ranking using data only from 10 randomly selected patients. (CSV 1053 kb)
