# Supplementary material for: Topconfects: a package for confident effect sizes in differential expression analysis provides a more biologically useful ranked gene list
Source: Genome Biol. 2019 Mar 28;20:67. doi: 10.1186/s13059-019-1674-7 (PMC6437914; doi:10.1186/s13059-019-1674-7)
Supplement: Supplementary file 1 — Additional figures. Additional figures giving more details on the simulation results. (PDF 2197 kb) [file 13059_2019_1674_MOESM1_ESM.pdf]

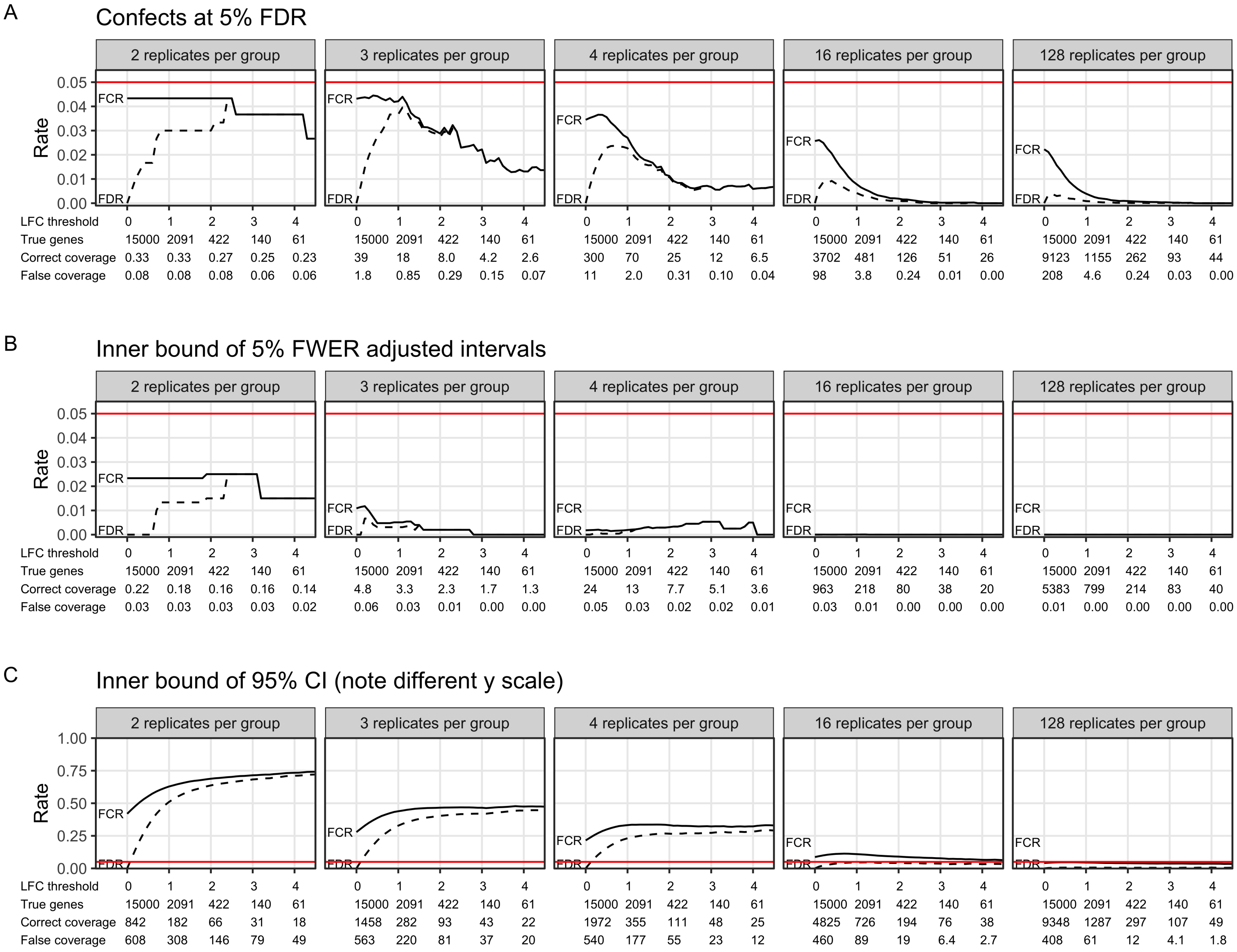

Figure S1. Comparison of achieved FDR and FCR by different methods for Simulation 1. The explanation of the graphs is the same as in Figure 1B. A.Confect confidence bounds as 5% FDR. B. Inner bounds derived from 5% FWER CIs. C. Inner bounds dervied from unadjusted 95% CIs.

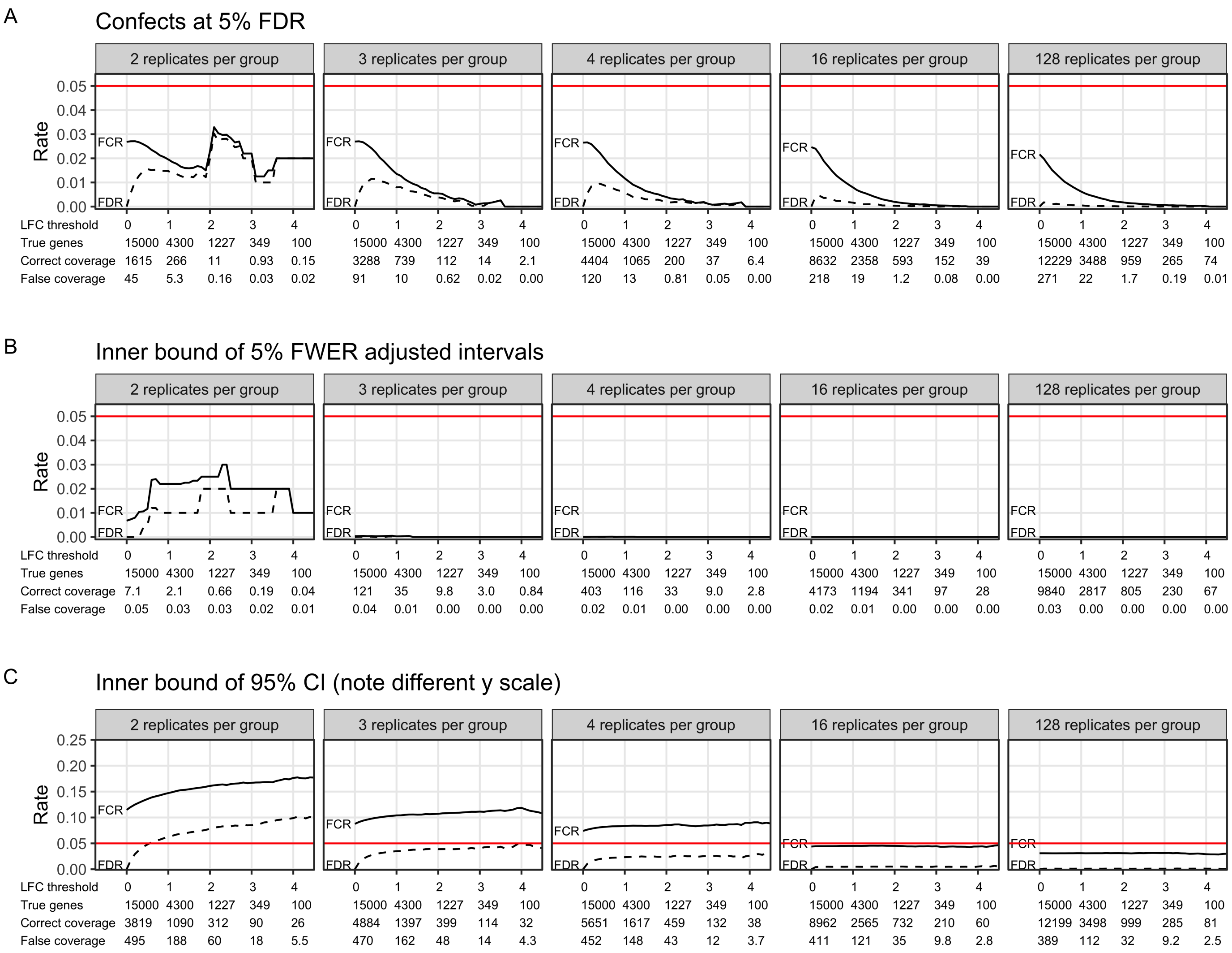

Figure S2. Comparison of achieved FDR and FCR by different methods for Simulation 2. The layout is the same as for Figure S1.

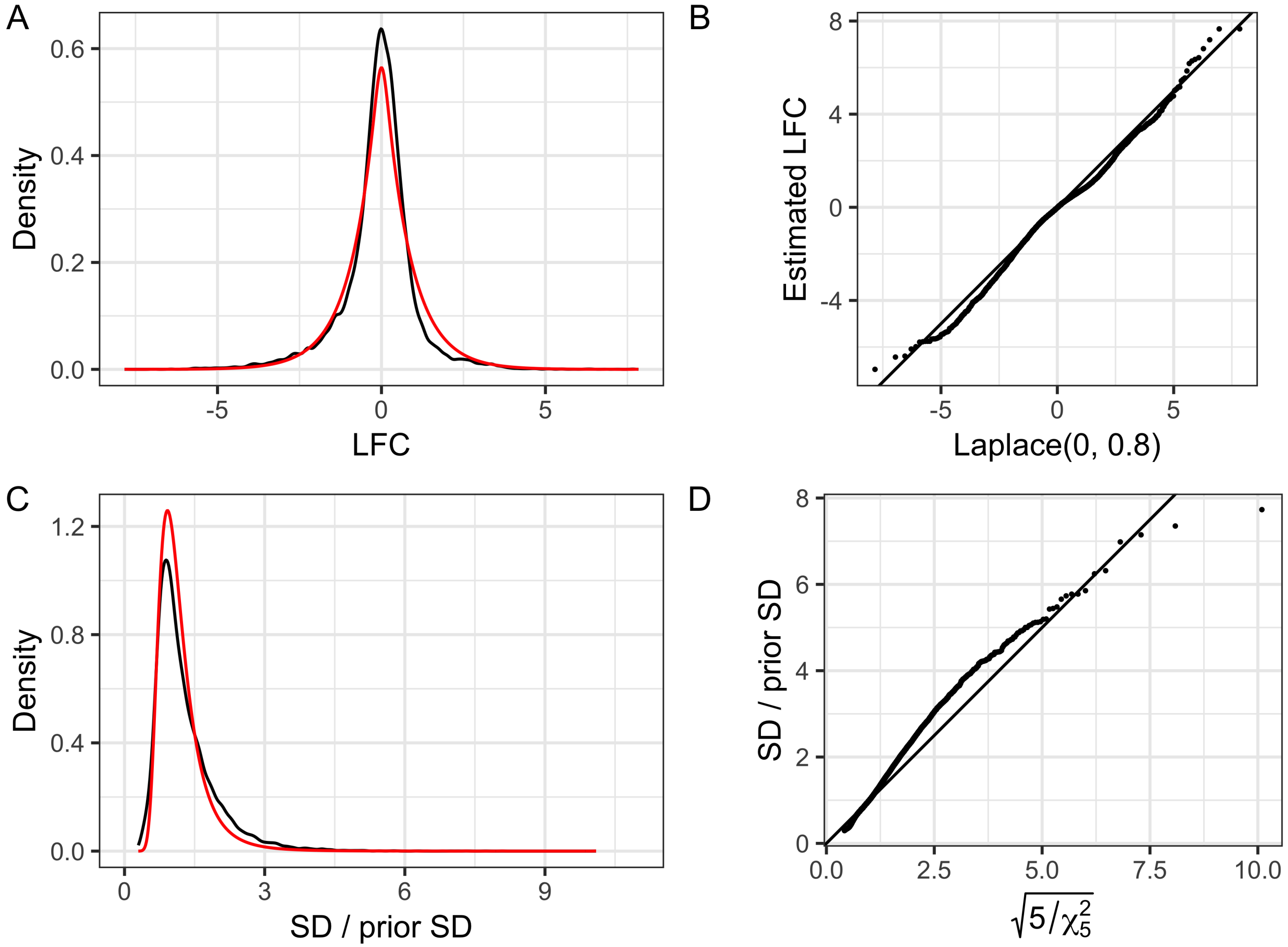

Figure S3. Estimated LFCs and residual standard deviations in the cancer data-set, and the distributions used to approximate these in Simulation 2. A. Density of estimated LFCs (black) and simulation distribution (red). B. Quantile-quantile plot comparing these two distributions. C. Density of standard deviations of the weighted residuals, after dividing out the empirical prior standard deviation. The distribution used in the simulation is shown in red. D. Quantile-quantile plot comparing these two distributions.
